# Prevalence and species identification of trematode metacercariae in Qiqihar, Northeast China

**DOI:** 10.3389/fmicb.2024.1464988

**Published:** 2024-09-10

**Authors:** Fengyu Zhang, Jianke Li, Shaocheng Zhang, Ting Chen, Hao Zhang

**Affiliations:** ^1^Department of Clinical Laboratory, Suining Central Hospital, Suining, China; ^2^School of Medical Technology, Qiqihar Medical University, Qiqihar, China; ^3^Department of Laboratory Medicine, The Second Affiliated Hospital of Chengdu Medical College (Nuclear Industry 416 Hospital), Chengdu, China; ^4^School of Clinical Medicine, Chengdu Medical College, Chengdu, China; ^5^Department of Pathology, Hospital of Chengdu Office of People’s Government of Tibetan Autonomous Region, Chengdu, China

**Keywords:** trematode metacercariae, phylogenetic evolution, prevalence, *Clonorchis sinensis*, *Metorchis orientalis*, *Metorchis taiwanensis*

## Abstract

Fishborne trematode (FBT) is an important group of parasites that are endemic worldwide to a certain extent. However, despite the epidemiological significance, the species and phylogenetic evolution characteristics of FBT metacercariae have not been well studied. In this study, a total of 600 *Pseudorasbora parva* (*P. parva*) specimens were collected from Qiqihar, 61.8% (371/600) were found to be infected with trematode metacercariae. A total of three kinds of trematodes metacercariae were obtained, and they were identified as *Clonorchis sinensis* (*C. sinensis*), *Metorchis orientalis* (*M. orientalis*), and *Metorchis taiwanensis* (*M. taiwanensis*) by morphological and phylogenetic analysis with infection rates of 47.7% (286/600), 15.5% (93/600), and 23.7% (142/600), respectively. Meanwhile, a survey of the three trematodes metacercariae showed that the infection rate of *C. sinensis* metacercariae was the highest in September, up to 66% (66/100), and the lowest in June at 26% (26/100). The infection rate of *M. orientalis* metacercariae was the highest in October at 26% (26/100) and the lowest in June at 5% (5/100). The infection rate of *M. taiwanensis* metacercariae was at its peak in November at 36% (36/100) and the lowest in July at 15% (15/100). The co-infection of metacercariae of *C. sinensis* and *M. taiwanensis* was the most common and reached a peak in October, and their infection rate was higher in autumn than in summer. The peak of infection intensity of metacercariae for *C. sinensis*, *M. orientalis,* and *M. taiwanensis* were different: *C. sinensis* was 24/g in September, *M. orientalis* was 7/g in October, and *M. taiwanensis* was 10/g in November. From the above results, it was confirmed that three species of trematodes metacercaria played an important role in infection of second intermediate hosts in Qiqihar region. Studying the morphological characteristics and sequencing the ITS2 gene for a phylogenetic tree of them will be useful for future molecular evolution, biology, and ecology of trematode metacercariae.

## 1 Introduction

Fishborne trematodiasis is a widespread parasitic disease, mainly distributed in Southeast Asian countries and regions (China, South Korea, Vietnam, Laos, etc.), with more than 12 million people infected (Sripa et al., 2021). Freshwater fish in China such as *Pseudorasbora parva*, *Carassius auratus,* and *Erythroculter ilishaeformi*, totaling 102 species in 59 genera and across 15 families act as the intermediate hosts of trematodes, including *Clonorchis sinensis* (*C. sinensis*), *Metorchis orientalis* (*M. orientalis*), *Metagonimus yokogawai* (*M. yokogawai*), and so on (Qiu et al., 2017; Tan and Machrumnizar, 2023). Hosts are commonly infected by consuming raw or undercooked fish meat containing metacercariae (Zhang et al., 2021). Adult parasites in hepatobiliary ducts or small intestine, such as *C. sinensis*, cause liver and bile duct damage or hepatic fibrosis (Wang et al., 2023). Therefore, *C. sinensis* is considered a group I carcinogen-metazoan parasite by the International Agency for Research on Cancer (IARC) that can potentially induce cholangiocarcinoma (Bouvard et al., 2009). As shown in former studies in China, from 2004 to 2012, the infection rate of *C. sinensis* in Guangdong Province was 8.6%, while in the survey of Guangxi from 2005 to 2014, the infection rate of *C. sinensis* was 9.9% (Lai et al., 2016). Even though it has epidemiological significance, the morphology and genetic variation studies of trematodes are still largely neglected (Huang et al., 2019).

Food-borne trematodes (FBTs) are taxonomically and phylogenetically diverse, many species are classified as zoonotic trematodes (Sohn et al., 2021). With the vigorous development of the fishery and aquaculture industry, the risk of exposure to parasites has increased (Duflot et al., 2021). Even if certain trematodes species, endemic areas, infection characteristics, and life habitats are well understood, they remain largely neglected due to a lack of taxonomic, epidemiological, and 8clinicopathological information on their species (Won et al., 2019; Brooker et al., 2010). In the present study, the prevalence, morphological, and phylogenetic characteristics of three trematode metacercariae will be evaluated through a relatively comprehensive analysis, this study will be crucial for understanding the prevalence of FBT understanding their molecular structure as well as providing suggestions to relevant departments for control strategies.

## 2 Materials and methods

### 2.1 *Pseudorasbora parva* collection and animal source

From June to November 2021, a total of 600 *P. parva* samples were collected from Qiqihar. *P. parva* were purchased from three markets (Jianhua District Market, Tiefeng District Market, and Longshan District Market) along the river and asked for the provenance where the fish were found. The collected fish were put in an ice bucket and brought back to the parasite laboratory of Qiqihar Medical College. Clean healthy Kunming mice and ducklings were fed in an SPF environment, provided by the Animal Laboratory Center of Qiqihar Medical University [Animal production license: SCXK (Liao) 20⁃0001; animal use license: SYXK (Black) 2021⁃013].

### 2.2 Purification of metacercariae and animal infection

All collected *P. parva* with ice were transferred to the laboratory (School of Medical Technology, Qiqihar Medical University, Qiqihar, China). Using the direct tablet pressing method, a size of 1 mm × 1 mm muscle was taken from the back, belly, and tail of the fish and was then placed between two glass slides, pressing firmly to find metacercariae under the microscope. The fish infected with metacercariae were judged to be positive. The positive fish were finely ground with a mortar with a pestle or a grinder. The ground fish meat was mixed with artificial gastric juice, and the mixture was incubated at 36°C for 12 h. The digested material was filtered with 1 mm × 1 mm of mesh and washed with 0.9% NaCl until the supernatant was clear. The sediment was carefully examined under a stereomicroscope. The metacercariae were separately collected and identified by their detailed morphological characteristics, and dimensions referred to relevant literature under a light microscope (Pakharukova et al., 2023; Kiyan et al., 2018). Identified metacercariae were stored at 4°C for later use, and some were experimentally infected in mice and ducklings to obtain adult worms. Each mouse and ducklings were infected with 100 metacercariae.

### 2.3 Genomic DNA extraction, amplification, and sequencing

The genomic DNA of the metacercariae was extracted using the DNeasy Blood & Tissue Kit (QIAGEN, Germany). The primers of the ITS2 gene were 3SF (5′-GGTACCGGTGGATCACTCGGCTCGTG-3′) and A28R (5′-GGGATCCTGGTTAGTTTCTTTTCCTCCGC-3′). Amplification of the ITS2 gene was applied following cycling conditions: 98°C for 10 s (denaturation), 58°C for 15 s (annealing), and 72°C for 5 s (extending), totaling 35 cycles. Negative controls with primers were included. TaKaRa Taq DNA polymerase was used for all the PCR amplifications. Then, the PCR products were separated in 1% agarose gel electrophoresis and visualized under UV light after staining with 4S GelRed. All the PCR products of the expected size were directly sequenced by Sangon Biotech Co., Ltd. (Shanghai, China). Each of the DNA products was approximately 520 bp.

### 2.4 DNA sequence analysis

All the nucleotide sequences in this study were aligned with each other. According to the identity percentage and query coverage parameter, the reference sequences were downloaded from the GenBank database using the Basic Local Alignment Search Tool (BLAST) to determine the isolates *Clonorchis sinensis*, *Metorchis orientalis,* and *Metorchis taiwanensis* in this study and representative nucleotide sequences acquired in the present study were deposited in the GenBank database under accession numbers ON287274, ON287358, and PP505449, respectively.

### 2.5 Evolutionary tree construction

To better present the diversity of all the isolates in this study and to estimate the genetic relationship of the novel sequences here to the known ones, intraspecific phylogenies were constructed with the neighbor-joining (NJ) and maximum-likelihood (ML) methods in the MEGA7.0 program. NJ trees were constructed using 1,000 bootstrap replicates. The Kimura 2-parameter “K2” model was used for the ML method. The sequences of KJ137227.1 (*C.sinensis*), MK450525.1 (*C.sinensis*), MW828640.1 (*C.sinensis*), AF217094.1 (*C.sinensis*), MT231323.1 (*M.orientalis*), MK482055.1 (*M.orientalis*), AY029182.1 (*Schistosoma hippopotami*), L03660.1 (*Schistosoma japonicum*), S72866.1 (*Schistosoma japonicum*), AB517580.1 (*Haplorchis taichui*), MT006051.1 (*H.taichui*), MH991969.1 (*H.taichui*), DQ351842.1 (*Fasciolopsis buski*), and MW771526.1 (*F.buski*) from GenBank were aligned with isolates from this study. *Taenia saginata* (AY954521.1) is an outgroup in this study.

### 2.6 Data analysis

IBM SPSS Statistics 26.0 was used for data analysis. The chi-square test was used to evaluate the assessment between qualitative variables to check for statistical differences. *p* < 0.05 was regarded as statistical significance.

## 3 Results

### 3.1 Infection rate of trematode metacercariae in *Pseudorasbora parva*

From June to November 2021, a total of 600 *P. parva* were collected, and 371 were found to be infected with trematode metacercariae, with an infection rate of 61.8%. A total of three kinds of trematode metacercariae were obtained as follows: metacercariae of *C. sinensis*, *M. orientalis*, and *M. taiwanensis* with infection rates of 47.7% (286/600), 15.5% (93/600) and 23.7% (142/600), respectively.

### 3.2 Morphological of trematode metacercariae

**Figure 1:**
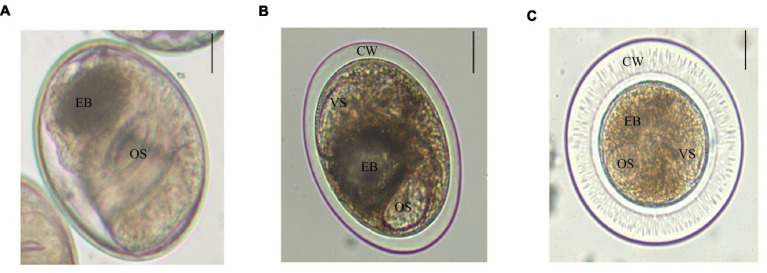
Three trematodes metacercariae in *P. parva*. Scale bar = 50 μm. **(A)** *C. sinensis* is elliptical and has two suckers, an oral sucker (OS) and a ventral sucker (VS), with browned excretory bladder (EB). **(B)** *M. orientalis* is elliptical, double cyst wall (CW), and an “O”-shaped excretory bladder. **(C)** *M. taiwanensis* has double thick cyst wall, and its oral sucker and ventral sucker are similar in size and with brownish excretory bladder.

There were three trematodes metacercariae in *P. parva*, numbered as metacercaria I, II, and III. Metacercariae I was elliptical, with a size of (0.149 ± 0.78) mm × (0.122 ± 0.94) mm. The oral and ventral suckers were about the same size. Double cyst walls and the inner wall were slightly thinner than the outer wall, approximately 3–4 μm. The excretory bladder was in an “O” shape, which was filled with brown particles. Larvae intermittently moved within the cyst wall (Figure 1A). Metacercariae II was elliptical, with a size of (0.156 ± 0.01) mm × (0.138 ± 0.01) mm. A double cyst wall, approximately 12 μm thick, O-shaped excretory bladder contains sepia particles (Figure 1B). Metacercariae III was globular or elliptical, with a size of (0.206 ± 0.02) mm × (0.203 ± 0.03) mm, transparent and very thick double cyst wall and with nearly equal-sized two suckers (Figure 1C). Careful identification of three trematodes metacercariae was checked out with reference to literature (Chai et al., 2012; Na et al., 2020). Metacercariae I, II, and III were initially identified as *Clonorchis sinensis*, *Metorchis orientalis,* and *Metorchis taiwanensis*, respectively.

### 3.3 Morphological of three adult worms

**Figure 2:**
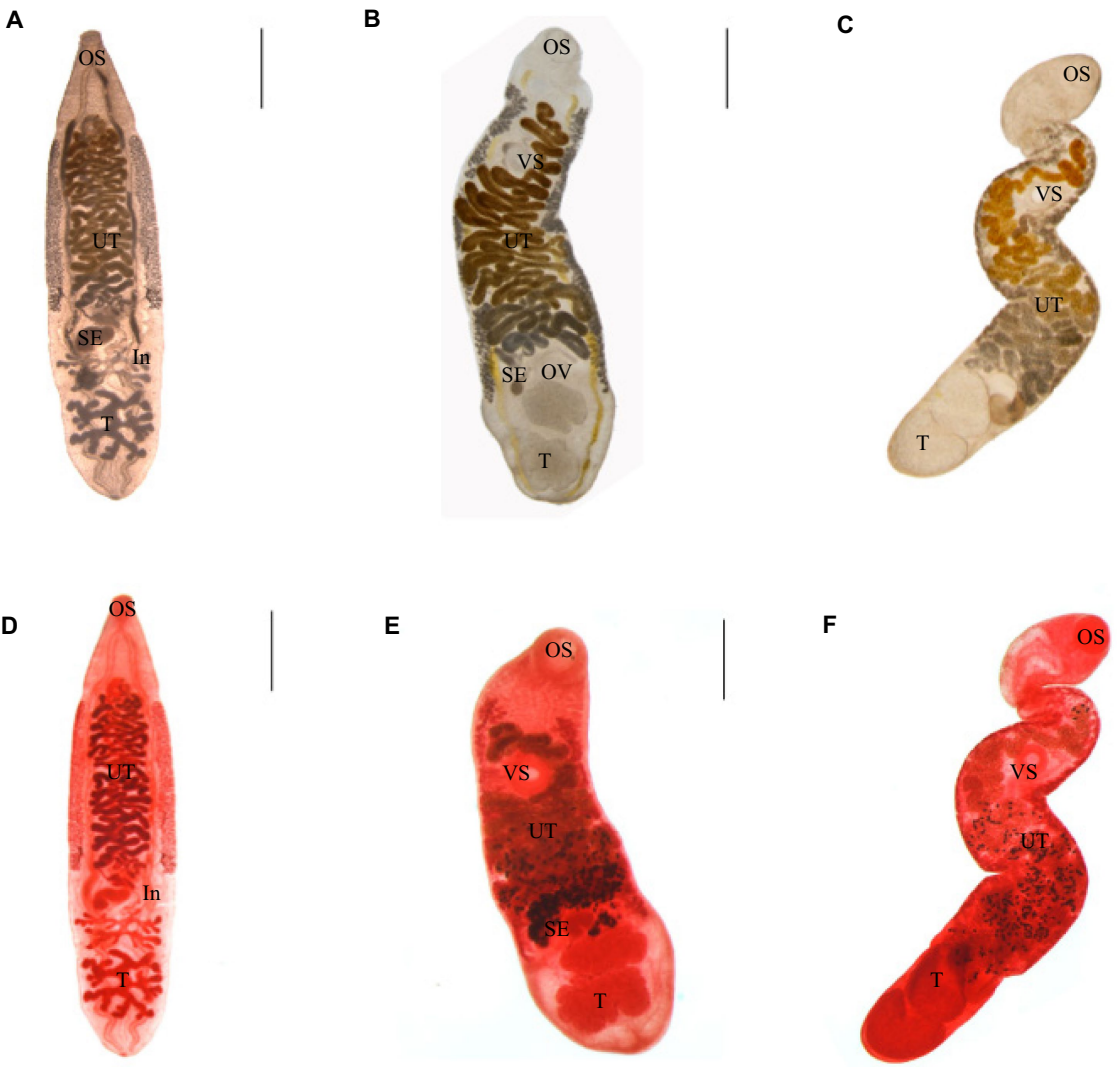
Three kinds of trematodes. **(A–C)** Adults of *C. sinensis*, *M. orientalis*, *M. taiwanensis*, live adults. **(D–F)** Acetocarmine stained adults. *C. sinensis* were recovered from mice. *M. orientalis* and *M. taiwanensis* recovered from ducklings. OS: oral sucker, *VS*: ventral sucker, UT: uterus, OV: ovary, SE: seminal vesicles, T: testicles, and In: intestines. Scale bar = 1 mm.

Adult worms infected with *C. sinensis* metacercariae parasitized the hepatobiliary ducts of mice. The adult was tabular, and the shape was like the kernel of sunflower seeds. They were (8.01 ± 2.04) mm × (1.93 ± 0.33) mm in average size; the oral sucker was located at the top of the body, nearly round, and the ventral sucker was located in the anterior 1/5 of the body. The two intestinal extended along both sides to the posterior of the body. The testis was highly branched, the anterior testis was divided into four branches, and the posterior testis was divided into five to six branches. The uterus was filled with eggs (Figures 2A,D).

Adult worms infected with *M. orientalis* metacercariae were found in the hepatobiliary ducts of ducks. The body was leaf-shaped, with a size of (5.35 ± 1.47) mm × (1.32 ± 0.36) mm in average size. The oral sucker was nearly round. The ventral sucker was located nearly 1/4 of the body. The testicles were lobes. The uterus began at the level of the ovaries and crossed the two intestinal tubes. The vitelline gland was granular and densely clustered, which was distributed on the outside of the two intestinal branches (Figures 2B,E).

Adult worms infected with *M. taiwanensis* metacercariae were obtained from the hepatobiliary ducts of ducks. The body was small and elongated, (4.04 ± 0.92) mm × (0.75 ± 0.29) mm covered with small spines on the surface of it. The oral and abdominal suctions were round. The fertilized vesicle was curved sac-shaped, located on the right side of the ovary, and had a ratio of 1.74:1 to ovarian length. The two testes are round or oval, arranged obliquely at the end of the body. The uterine began at the level of the ovaries and reached between the intestinal branch and the ventral sucker (Figures 2C,F).

### 3.4 Sequencing and phylogenetic analysis of ITS2 gene of three trematode metacercarias

First, based on morphological characteristics, three kinds of metacercariae were preliminarily identified as the metacercariae of *C. sinensis*, *M. orientalis,* and *M. taiwanensis*. PCR amplification and sequencing verification of metacercariae species were performed using the ITS2 gene. The results of 1% agarose gel electrophoresis of PCR products showed that three fragments of about 520 bp appeared in the lane, and there was no non-specific band (Supplementary Figure S1).

The lengths of ITS2 sequences obtained from PCR products of *C. sinensis*, *M. orientalis,* and *M. taiwanensis* were 533 bp, 524 bp, and 522 bp, respectively (Supplementary Figure S2).

The obtained sequences were compared with NCBI BLAST results.^1^ The results revealed that all sequences obtained in this study were 98–100% identity at the highest with one of the deposited sequences of trematode species in GenBank (Table 1).

**Table 1:** Identification of ITS2 gene sequences of trematodes metacercariae isolates from *P. parva* in Qiqihar using the NCBI BLAST search.

	Length (BP)	GenBank accession no.	Identity (100%) and remarks	Isolation resource	Location
*C.sinensis*	533	ON287274		Fish	This study
		KJ137227.1	506/506 (100%)	Fish	Nanning, Guangxi
		MW828640.1	527/529 (99.6%)	Feces	Changchun, Jilin
		MK450525.1	527/529 (99.6%)	Dog	Daqing, Heilongjiang
		AF217094.1	496/497 (99.8)	Fish	Kimhae, South Korea
*M.orientalis*	524	ON287358		Fish	This study
		MT231323.1	515/520 (99.0%)	Black swan	Changchun, Jilin
		MK482055.1	515/521 (98.8%)	Duck	Daqing, Heilongjiang
*M.taiwanensis*	522	PP505449		Fish	This study

**Figure 3:**
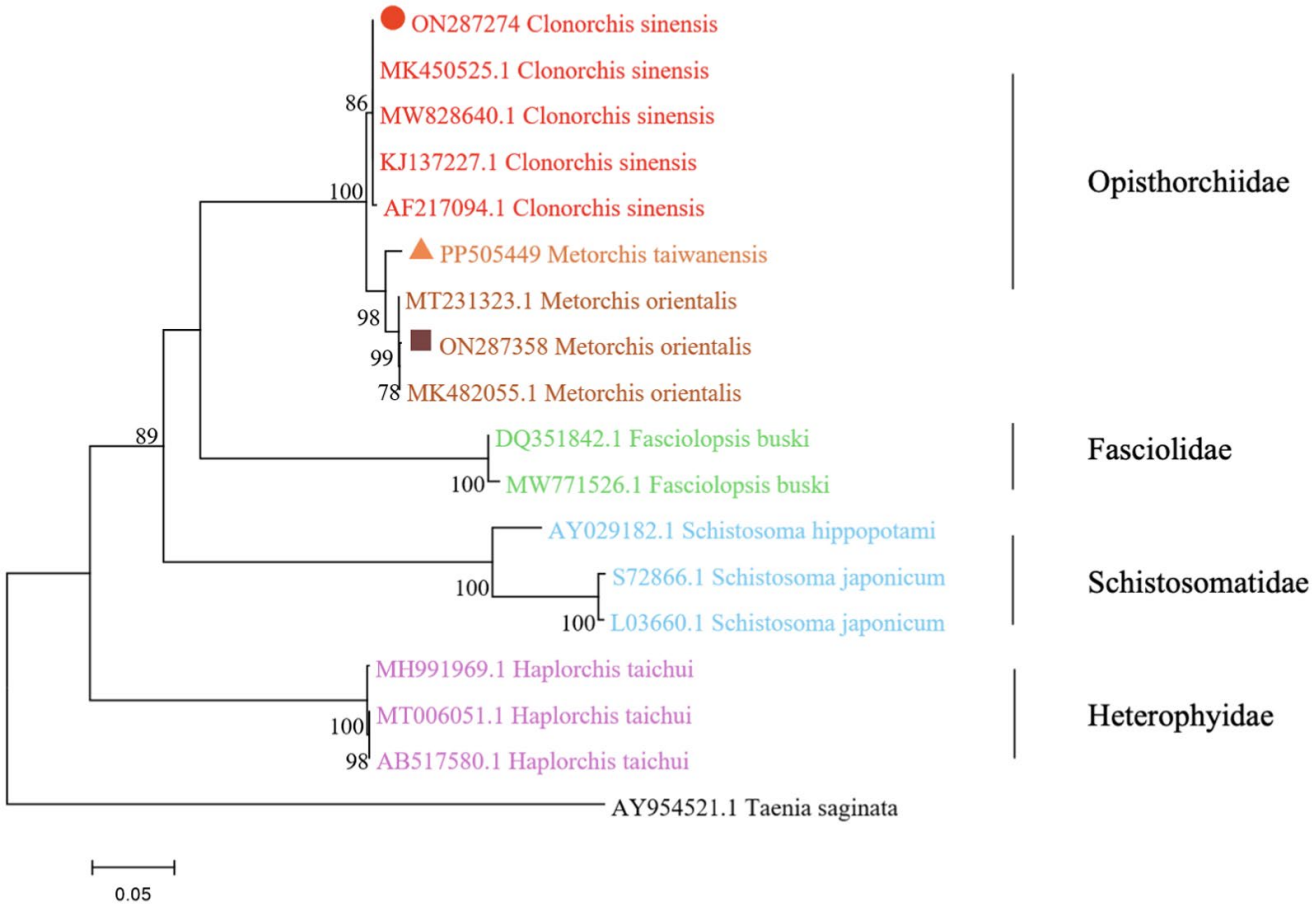
Phylogenetic relationships between the *C. sinensis*, *M. orientalis,* and *M. taiwanensis* isolates based on the ITS2 rDNA sequences using the neighbor-joining (NJ) method. Schistosoma hippopotami and Haplorchis taichui were used for comparison. Each sequence was identified by its accession number. Genotypes with black triangle were isolates identified in this study, respectively.

**Figure 4:**
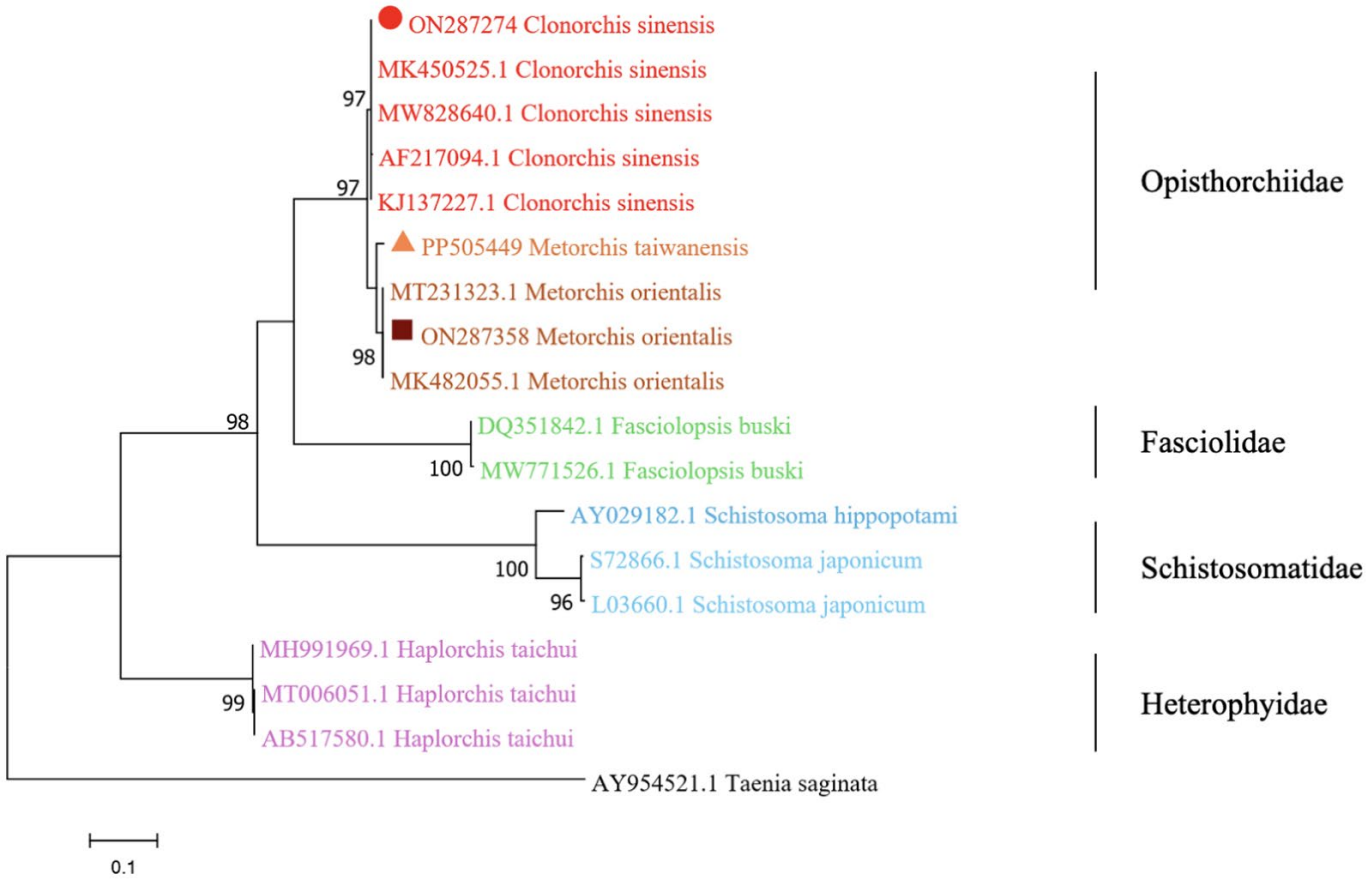
Phylogenetic relationships between the *C. sinensis*, *M. orientalis,* and *M. taiwanensis* isolates based on the ITS2 rDNA sequences using the maximum-likelihood (ML) method. Schistosoma hippopotami and Haplorchis taichui were used for comparison. Each sequence is identified by its accession number. Genotypes with black triangles are isolates identified in this study, respectively.

The phylogenetic analysis was conducted based on the ITS2 gene sequences of the isolates of *M. orientalis*, *C. sinensis*, and *M. taiwanensis* in this study and some isolates published on GenBank, inferred by neighbor-joining (NJ) and maximum likelihood (ML) trees when the sequence of *Taenia saginata* (AY954521.1) was used as an outgroup. As shown in Figures 3, 4, the topologies of NJ and ML trees were very similar and only small differences in bootstrap values were accessed among those isolates. The trees revealed that the trematodes ITS2 sequences were divided into four families, the family Opisthorchiidae, Fasciolidae, Schistosomatidae, and Heterophyidae. Meanwhile, the phylogenetic tree shows that the *C. sinensis*, *M. orientalis*, and *M. taiwanensis* form a large topological branch, while the *Taenia saginata* were independent of this large branch. The metacercaria of *M. orientalis*, *C. sinensis,* and *M. taiwanensis* in this study, as well as the *M. orientalis* and *C. sinensis* downloaded from the database, form small topological branches with bootstrap values of more than 95%.

### 3.5 Infection rate of three trematodes metacercariae in *Pseudorasbora parva*

**Figure 5:**
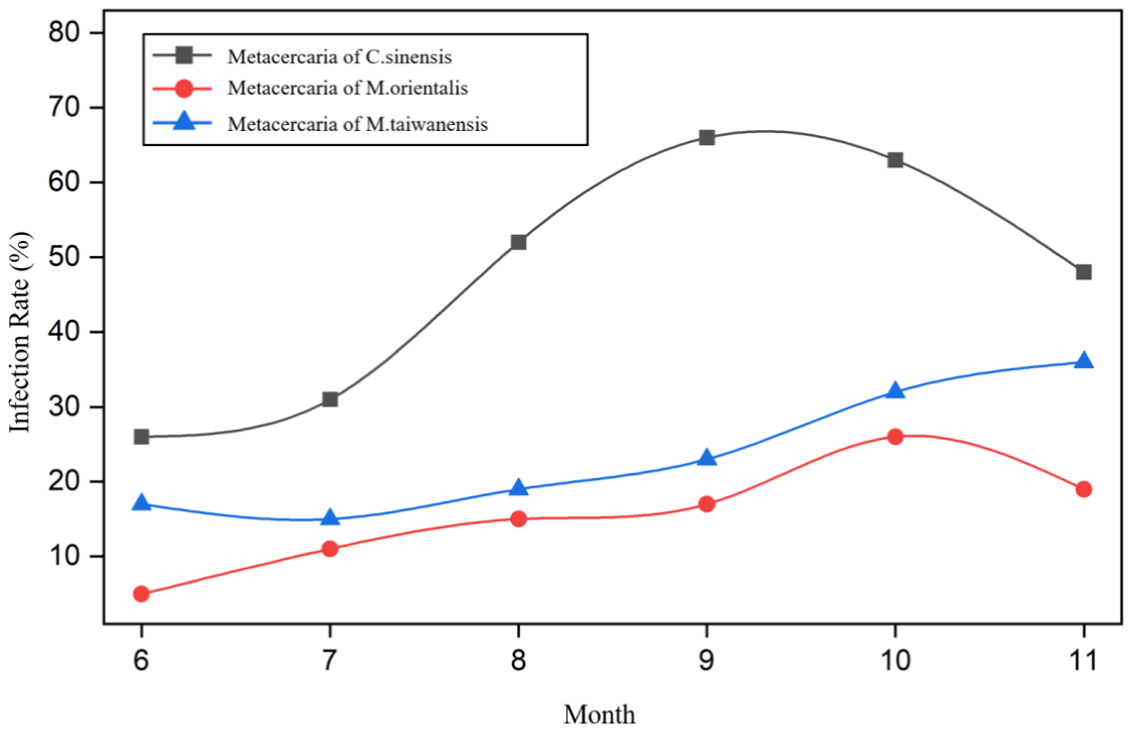
Trend of *P. parva* infected with trematodes metacercariae in different months.

From June to November 2021, the infection rates of metacercariae of *C. sinensis* in *P. parva* were 26.0% (26/100), 31.0% (31/100), 52.0% (52/100), 66.0% (66/100), 63.0% (63/100), and 48.0% (48/100), respectively. The infection rates of metacercaria of *M. orientalis* were 5.0% (5/100), 11.0% (11/100), 15.0% (15/100), 17.0% (17/100), 26.0% (26/100), and 19.0% (19/100), respectively. The infection rates of metacercaria of *M. taiwanensis* were 17.0% (17/100), 15.0% (15/100), 19.0% (19/100), 23.0% (23/100), 32.0% (32/100), and 36.0% (36/100), respectively (Figure 5; Supplementary Table S1). There were statistically significant differences in infection rates among the three types of metacercariae in different months (*χ*^2^ = 53.61, 19.51, 20.11, all *p* < 0.05).

The prevalence of metacercariae of *C. sinensis*, *M. orientalis,* and *M. taiwanensis* in summer were 36.3% (109/300), 10.3% (31/300), and 17.0% (51/300) while in autumn were 59.0% (177/300), 20.7% (62/300), and 30.3% (91/300), respectively (Supplementary Table S2). There were significant differences in the infection rates between the three trematodes metacercariae in summer and autumn (*χ*^2^ = 30.89, 12.23, 14.71, all *p* < 0.05). Meanwhile, there were statistically significant differences in infection rates among the three species in summer and autumn (*χ*^2^ = 22.29, 33.96, all *p* < 0.05).

From June to November, the infection intensity of metacercariae of *C. sinensis* in *P. parva* was 6, 6, 13, 24, 14, and 5 (average gram), respectively. The corresponding infection intensity of metacercaria of *M. orientalis* in *P. parva* was 1, 1, 3, 5, 7, and 4 (average gram) respectively. *M.taiwanensis* was 2, 3, 5, 4, 6, and 10 (average gram) respectively. (Supplementary Table S3).

### 3.6 Co-infection rate of three trematodes metacercariae in *Pseudorasbora parva*

From June to November 2021, the co-infection rates of metacercariae of *C. sinensis* and *M. orientalis* in *P. parva* were 2.0% (2/100), 4.0% (4/100), 7.0% (7/100), 9.0% (9/100), 15.0% (15/100), and 9.0% (9/100), respectively. The co-infection rates of metacercariae of *C. sinensis* and *M. taiwanensis* were 7.0% (7/100), 6.0% (6/100), 9.0% (9/100), 11.0% (11/100), 18.0% (18/100), and 15.0% (15/100), respectively. The co-infection rates of metacercariae of *M. orientalis* and *M. taiwanensis* were 0% (0/100), 1.0% (1/100), 2.0% (2/100), 3.0% (3/100), 7.0% (7/100), and 5.0% (5/100), respectively. The co-infection rates of metacercariae of *C. sinensis*, *M. orientalis,* and *M. taiwanensis* were 0% (0/100), 0% (0/100), 0% (0/100), 1.0% (1/100), 3.0% (3/100), and 1.0% (1/100), respectively. (Supplementary Table S4). There were statistically significant differences in co-infection rates between the two types of metacercariae in different months (*χ*^2^ = 14.60, 11.16, 11.68, all *p* < 0.05). However, there were no statistically significant differences in co-infection rates among the three types of metacercariae in different months (*χ*^2^ = 1.627, *p* > 0.05).

The co-infection rate of metacercariae of *C. sinensis* and *M. orientalis* in summer and autumn were 4.3% (13/300) and 11.0% (33/300). The co-infection rate of metacercariae of *C. sinensis* and *M. taiwanensis* in summer and autumn were 7.3% (22/300) and 14.7% (44/300). The co-infection rate of metacercariae of *M. orientalis* and *M. taiwanensis* in summer and autumn were 1.0% (3/300) and 5.0% (15/300). The co-infection rates of metacercaria of *C. sinensis*, *M. orientalis,* and *M. taiwanensis* in summer and autumn were 0% (0/300) and 1.7% (5/300), respectively. (Supplementary Table S5). There were statistically significant differences in co-infection rates between the three types of metacercariae in summer and autumn (*χ*^2^ = 9.42, 8.24, 8.25, 5.04, all *p* < 0.05).

## 4 Discussion

FBT causes a huge number of threats to humans and poultry (ducks, chicken, and geese) and other mammals such as dogs and cats (Won et al., 2019). There exists many varieties, ranging in awareness around the world (Yoo et al., 2022). Due to a large number of trematodes parasites in fish and the absence of corresponding epidemiological and species information, these trematodes have been hugely neglected (Labony et al., 2020). Therefore, the species identification and molecular characteristics of metacercariae are of great significance (Ma et al., 2024).

There have been many reports of trematodes in fish, and a variety of fish species can be used as intermediate hosts (Chai and Jung, 2017). In this study, three different types of metacercariae were found and compared with former literature (Sohn et al., 2009); ultimately, they were identified as *C. sinensis*, *M. orientalis,* and *M. taiwanensis* by infecting animals and ITS2 gene sequencing. The monthly survey of three kinds of trematodes metacercariae showed that their infection rate fluctuated from June to November and that it was higher in autumn than in summer. At present, there are many studies on the infection of trematode metacercariae in fish, but few focus on the seasonal dynamics of them (Qiu et al., 2017; Hung et al., 2013). The peak of trematode metacercariae infection was in autumn, which was slightly different from the peak of infection in the summer in former study (Zhang et al., 2019). We speculated that the time it took for the trematode metacercariae to mature was prolonged, so the number of them began to multiply in the summer and reach to peak in the autumn. In this present study, the peak of infection intensity of metacercariae of three kinds of trematode metacercariae were very different: *C. sinensis* was 24/g in September, *M. orientalis* was 7/g in October, and *M. taiwanensis* was 10/g in November, the month with the highest infection rate was simultaneously the highest intensity of infection. We also analyzed the co-infection of trematode metacercariae in *P. parva* from June to November, and the co-infection of *C. sinensis* and *M. taiwanensis* was the most common and reached a peak in October, with three metacercariae co-infected exists, but relatively rare. In the seasonal analysis, the co-infection of *C. sinensis* and *M. taiwanensis* was the most common, and the infection rate was higher in autumn than in summer. This is the first time that infection of metacercariae of *M. taiwanensis* has been investigated in Qiqihar. Furthermore, in order to verify the species of trematode metacercariae, adults of three trematodes were recovered from infected animals and according to their morphological characteristics and internal organ measurements, compared with the former study (Wang et al., 2020), the three adult trematodes belonged to the family Opisthorchiidae, the genus of *Clonorchis* and *Metorchis*.

ITS rDNA spacers usually diverge among species but are homogeneous within species due to concerted evolution (Yang et al., 2024; Beesley et al., 2023). Based on the analysis of the ITS2 region, this was the first genetic analysis of *M. taiwanensis* from freshwater fish in Qiqihar. The results could act as a significant reference for future studies on *M. taiwanensis*, including species identification and evaluation of molecular variations between separate geographical locations. *C. sinensis* were successfully identified by amplification using the ITS gene (Oh et al., 2022). In the same way, the ITS2 gene has also been reported as a molecular marker in the analysis between *Opisthorchis felineus* and the genus *Clonorchis* spp. (Kiyan et al., 2018). Amplifying the ITS1 and ITS2 gene sequences and constructed a phylogenetic tree, which matched the identification results of morphological characteristics, indicating that the identification of species by ITS amplification sequencing and species by morphological characteristics supporting the same taxonomic classification (Poulin et al., 2019; Xie et al., 2024).

As is shown in Figures 3, 4, *C. sinensis* (KJ137227.1 from Nanning; MW8286640.1 from Changchun; MK450525.1 from Daqing) were clustered with isolates from *C. sinensis* in this present study and they formed a large branch with *C. sinensis* isolated from Korea, indicating that there were no different branches due to geographical differences and variations of individual bases during phylogenetic tree analysis (Bawm et al., 2022). Meanwhile, it also shows the ITS2 might be an appropriative and sensitive marker for species-level analysis. The isolates *M. orientalis* in the present study are clustered together with the *M. orientalis* (MT231323.1 from Changchun; MK482055.1 from Daqing), which is consistent with the former study (Li et al., 2016). In addition, *M. orientalis* and *M. taiwanensis* were in the same topological branch with a 95% bootstrap value in the phylogenetic tree constructed with the NJ method, demonstrating that they are closely related. Furthermore, the phylogenetic tree shows three kinds of metacercariae were on a large topological clade, suggesting that it may have evolved from the same ancestor and also verifies the correctness of morphological judgment from the side. What’s more, such information can be also beneficial to improve our understanding of the molecular mechanisms of species adaptation and evolution and parasite infection strategies (Dufour et al., 2013).

In this study, due to the lack of *M. taiwanensis* genome sequencing information on GenBank, effective alignment could not be well performed. However, it can be seen from the constructed phylogenetic tree that it is still in the same branch as *M. orientalis*, indicating that *M. taiwanensis* is closely related to *M. orientalis*. Based on the analysis of ITS2 region from GenBank sequences, this was the first analysis of ITS2 sequences of *M. taiwanensis* as well as prevalence investigation from *P. parva* in Qiqihar, Heilongjiang Province. Research could provide an important reference for future studies on *M. orientalis*, *C. sinensis,* and *M. taiwanensis* whether it is morphological identification or the assessment of molecular variation between diverse geographical locations. More significantly, our study will be accessed for the further classification and identification of FBT trematodes, providing methodologies suitable for disease prevention and control strategies.

## Data Availability

The datasets presented in this study can be found in online repositories. The names of the repository/repositories and accession number(s) can be found in the article/Supplementary material.

## Footnotes

^1^ https://blast.ncbi.nlm.nih.gov/Blast.cgi
