# Supplementary material for: Prevalence and species identification of trematode metacercariae in Qiqihar, Northeast China
Source: Front Microbiol. 2024 Sep 10;15:1464988. doi: 10.3389/fmicb.2024.1464988 (PMC11420014; doi:10.3389/fmicb.2024.1464988)
Supplement: Supplementary file 1 [file Data_Sheet_1.pdf]

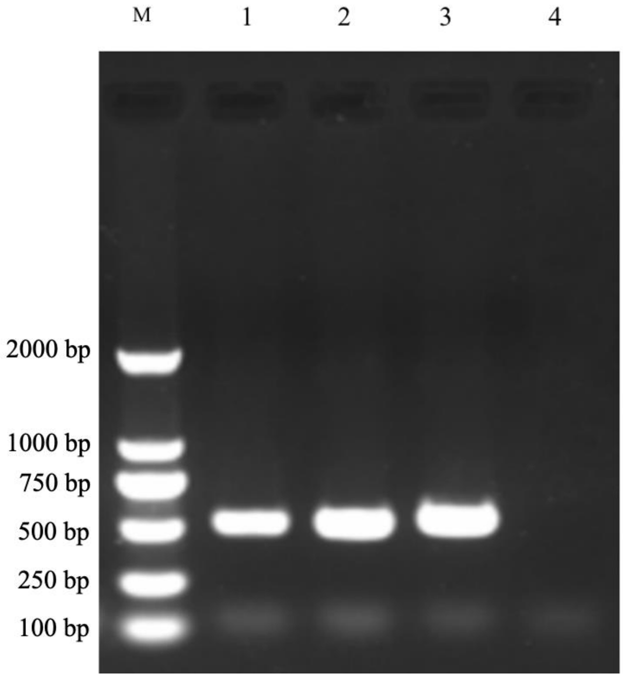

Figure S1
PCR amplification electrophoresis of ITS2. M: DNA marker. 1: C. sinensis 2: M. orientalis 3: M. taiwanensis 4: Negative control

A

GGGATCCTGGTTAGTTTCTTTTCCTCCGCTTAGTGATATGCTTAAGTTCAGC
GGGTAATCACGTCTGATCCGAGGTCAGGAAAGTTAAGCACCGACCGGTGC
AAAACAGATTTGCATCGAATGCATTGCCAATACTGAAGCCTCAACCAAAGA
CAAAGGACCAACAACGGAGCGCGCACATTCACAACAATAACAACAATTGA
GCCACGACTCCGCCGCCACCCCCTCATCTAGGCAGTCAGCCCAGACATGGT
TGCGTCCGGCACATTGGGGAAAAGCCATAGATCCGGCACCCCACACACATA
CACACAATTGTGTGGGGAAATCATGCCAGCTGGCAAGACCCAAGCCACGA
CTTTTTGGGCGTCGTGATAGTTTATAAGCCGACCCTCGGACAGGCGTGGCC
ACAGGCAAACCCATGGCCGCAATATGCGTTCAAGATGTCGATGTTCAAAGC
AGTATGCAGTTCGCATTAATTCACACAGTTGGCTGCGCTCTTCATCGACACA
CGAGCCGAGTGATCCACCGGTACC

B

GGGATCCTGGTTAGTTTCTTTTCCTCCGCTTAGCTGATGCATGCTTAAGTTC
AGCGGGTAATCACGTCTGATCCGAGGTCAGGAAAGTTAAGCACGGAAG
TGCAAAACAGATTTGCATCGAATGCATTGCCACTACTGAAGCCTCAACCA
AAGACAAAGGACCAACAACGGAGCGCGCACATCCACAACAATAAGAACA
ATTGAGCCACGACTCCGCCGCCACCCCCTCATCTAGGCAGTCAGCCCAGAC
ATGGTTGCGTCCGGCACATTGGGGAAAAGCCACAGATCCGGCACCCCACA
CAAAAGTGCGAGGAAATCATGCCAGCTGGCAAAACCCAAGCCACGACTTT
TTGGGCGTCGTGATAGTTTATAAGCCGACCCTCGGACAGGCGTGGCCACAG
GCAAACCCATGGCCGCAATATGCGTTCAAGATGTCGATGTTCAAAGCAGTA
TGCAGTTCGCATTAATTCACACAGTTGGCTGCGCTCTTCATCGACACACGA
GCCGAGTGATCCACCGGTACC

C

GGGATCCTGGTTAGTTTCTTTTCCTCCGCTTAGCTGATGCATGCTTAAGTTC
AGCGGGTAATCACGTCTGATCCGAGGTCAGGAAAGTTAAGCACGGAAG
TGCAAAACAGATTTGCATCGAATGCATTGCCACTACTGAAGCCTCAACCA
AAGACAAAGGACCAACAACGGAGCGCGCACATCCACAACAATAAGAACA
ATTGAGCCACGACTCCGCCGCCACCCCCTCATCTAGGCAGTCAGCCCAGAC
ATGGTTGCGTCCGGCACATTGGGGAAAAGCCACAGATCCGGCACCCCACA
CAAAAGTGCGAGGAAATCATGCCAGCTGGCAAAACCCAAGCCACGACTTT
TTGGGCGTCGTGATAGTTTATAAGCCGACCCTCGGACAGGCGTGGCCACAG
GCAAACCCATGGCCGCAATATGCGTTCAAGATGTCGATGTTCAAAGCAGTA
TGCAGTTCGCATTAATTCACACAGTTGGCTGCGCTCTTCATCGACACACGA
GCCGAGTGATCCACCGGTACC

Figure S2
The sequence of three kinds of trematode metacercaria. **(A)** *C. sinensis*. 533bp. **(B)** *M. orientalis.* 524bp. **(C)** *M. taiwanensis.* 522bp

TABLE S1 Infection rates of trematodes metacercaria in *P. parva* in different months.

Month	Infection rates (%)		
	C. sinensis	M. orientalis	M. taiwanensi
6	26	5	17
7	31	11	15
8	52	15	19
9	66	17	23
10	63	26	32
11	48	19	36

TABLE S2 Comparison of infection rates of three trematodes metacercaria in *P. parva* in Summer and Autumn

season	Infection rates (%)		
	C. sinensis	M. orientalis	M. taiwanensis
summer	36.3	10.3	17.0
autumn	59.0	20.7	30.3

TABLE S3 Comparison of infection intensity of three trematodes metacercaria in P. parva in different month.

month	Intensity of infection		
	C.sinensis	M.orientalis	M.taiwanensis
6	6	1	2
7	6	1	3
8	13	3	5
9	24	5	4
10	14	7	6
11	5	4	10

TABLE S4 Co-infection rates of trematodes metacercaria in *P. parva* in different months.

Month	Co-infection rates (%)			
	C. sinensis and M. orientalis	C. sinensis and M. taiwanensis	M. orientalis and M. taiwanensis	C. sinensis, M. orientalis and M. taiwanensis
6	2	7	0	0
7	4	6	1	0
8	7	9	2	0
9	9	11	3	1
10	15	18	7	3
11	9	15	5	1

TABLE S5 Co-infection rates of three trematodes metacercaria in *P. parva* in Summer and Autumn

season	Co-infection rates (%)			
	C. sinensis and M. orientalis	C. sinensis and M. taiwanensis	M. orientalis and M. taiwanensis	C. sinensis, M. orientalis and M. taiwanensis
summer	4.3	7.3	1	0
autumn	11	14.7	5	1.7
